# Editorial: Biomedical informatics applications in rare diseases

**DOI:** 10.3389/frai.2022.1051182

**Published:** 2022-10-27

**Authors:** Qian Zhu, Yong-Zi Chen, Yanji Xu

**Affiliations:** ^1^Division of Pre-Clinical Innovation, National Center for Advancing Translational Sciences (NCATS), Rockville, MD, United States; ^2^Laboratory of Tumor Cell Biology, Tianjin Medical University Cancer Institute and Hospital, Tianjin, China; ^3^Division of Rare Diseases Research Innovation, National Center for Advancing Translational Sciences (NCATS), Bethesda, MD, United States

**Keywords:** rare disease (RD), clinical decision support (CDS), natural language processing (NLP), data integration, knowledge graph (KG)

A rare disease is defined in the US as a disease affecting <200,000 people. It is estimated that 400 million people globally are affected by rare diseases, and it takes an average of 5 years for rare disease patients to receive an accurate diagnosis. Due to diagnostic delay and lack of effective therapeutic interventions, 30% of children with a rare disease are not expected to see their fifth birthday, while nearly 95% of rare diseases lack an FDA-approved treatment. On the other hand, ever-increasing amounts of data collected and managed for genetics and biomedical evidence present new opportunities for breakthroughs to improve diagnosis and treatments in rare diseases. Advanced biomedical informatics approaches hold great promise to support diagnosis, drug discovery, and clinical trials in rare diseases. The ability of computational technologies to identify novel patterns in data, particularly data from different sources (e.g., multi-omics, patient registries, and so on), can be applied to overcome current challenges including poor diagnostic rates, lack of treatment standards, misunderstood etiology, and so on. Innovations in science, technology, and the use of big data and AI analytics are enabling great leaps forward in rare disease knowledge and treatment.

This Research Topic contains five papers, including three papers that are extended versions of the original full-length papers presented at a BIBM workshop in 2021. These papers cover a wide range of Research Topics and their applications in biomedical informatics research for rare diseases, by introducing novel computational technical solutions and emerging integrative clinical and biomedical resources developed to support scientific research and clinical decision-making in rare diseases. Each of the following five articles made a novel and complementary contribution to this Research Topic.

Brown et al. introduced a first feasibility study on using a wearable biosensor to detect heart rate variability (HRV), a physiological marker of sympathovagal balance, in Amyotrophic Lateral Sclerosis (ALS) patients. Cardiovascular dysautonomia may impact the quality of life and survival in ALS. Such dysfunction is not systematically assessed in these patients. Wearable devices could help address this. The preliminary results suggest that remote HRV measures using VitalConnect is feasible and may constitute an improved strategy to provide insights into sympathovagal balance in ALS.

Foksinska et al. introduced an artificial intelligence tool, mediKanren, that enables an efficient way to link all relevant literature and databases in the form of knowledge graphs. mediKanren leverages the mechanistic insight of genetic disorders to identify therapeutic options and has allowed for a scalable process that has been used to help over 500 rare disease families. In this paper, they provide a description of their process, the advantages of mediKanren, and its impact on rare disease patients.

Karas et al. detailed their comparable experiments with LDA and Top2Vec for embedded topic discovery on social media data. In this study, they aimed at experimenting with LDA model optimization and examination of the Top2Vec model with different embedding models for topic modeling. From their experiments, it illustrated that the Top2Vec model with doc2vec as the embedding model has higher coherence and qualitatively higher human readability of derived topics, demonstrated with a case study of Cystic Fibrosis. The generalizable workflow will be expanded to other types of social media data for other rare diseases for better assessing patients' needs from social media data.

Fecho et al. leveraged the Integrated Clinical and Environmental Exposures Service (ICEES) as an open electronic health record data and environmental exposures data to derive insights into rare pulmonary disease. They described a proof-of-concept application of ICEES to examine demographics, clinical characteristics, environmental exposures, and health outcomes among a cohort of patients enriched for phenotypes associated with cystic fibrosis (CF), idiopathic bronchiectasis (IB), and primary ciliary dyskinesia (PCD). We replicate current understanding of the pathogenesis and clinical manifestations of CF by identifying co-diagnoses of asthma, chronic nasal congestion, cough, middle ear disease, and pneumonia as factors that differentiate patients with poor health outcomes from those with better health outcomes.

Zhu et al. introduced a rare disease-based scientific annotation knowledge graph. A relatively large volume of RD-related publications accumulated in recent years and offer opportunities to utilize these publications for accessing the full spectrum of the scientific research and supporting further investigation in RD. In this study, they systematically analyzed, semantically annotated, and scientifically categorized RD-related PubMed articles, and integrated those semantic annotations in a knowledge graph (KG), which is hosted in Neo4j based on a predefined data model. With the successful demonstration of scientific contribution in RD *via* the case studies performed by exploring this KG, they proposed to extend the current effort by expanding more RD-related publications and other types of resources as a next step.

## Author contributions

QZ, Y-ZC, and YX were guest associate editors of the Research Topic. QZ drafted the manuscript. All authors have made a substantial contribution to the Research Topic and approved the manuscript for publication.

## Funding

This research was supported in part by Intramural Research Program (1ZIATR000410–03) and the extramural research program of the NCATS, NIH.

## Conflict of interest

The authors declare that the research was conducted in the absence of any commercial or financial relationships that could be construed as a potential conflict of interest.

## Publisher's note

All claims expressed in this article are solely those of the authors and do not necessarily represent those of their affiliated organizations, or those of the publisher, the editors and the reviewers. Any product that may be evaluated in this article, or claim that may be made by its manufacturer, is not guaranteed or endorsed by the publisher.

